# Elucidating prognosis in cervical squamous cell carcinoma and endocervical adenocarcinoma: a novel anoikis-related gene signature model

**DOI:** 10.3389/fonc.2024.1352638

**Published:** 2024-06-26

**Authors:** Mingwei- Wang, Qiaohui- Ying, Ru Ding, Yuncan- Xing, Jue Wang, Yiming- Pan, Bo Pan, Guifen- Xiang, Zhong Liu

**Affiliations:** ^1^ Institute of Blood Transfusion, Chinese Academy of Medical Sciences and Peking Union Medical College, Chengdu, China; ^2^ Institute of Oral Basic Research, School and Hospital of Stomatology, Cheeloo College of Medicine, Shandong University, Jinan, China; ^3^ Department of Obstetrics and Gynecology, The First Hospital of Jilin University, Changchun, China; ^4^ National Cancer Center/National Clinical Research Center for Cancer/Cancer Hospital, Chinese Academy of Medical Sciences and Peking Union Medical College, Beijing, China; ^5^ School of Public Health, Anhui Medical University, Hefei, China

**Keywords:** cervical cancer, anoikis-related genes, immune microenvironment, decision curve analysis, prognostic nomogram

## Abstract

**Background:**

Cervical squamous cell carcinoma and endocervical adenocarcinoma (CESC) are among the most prevalent gynecologic malignancies globally. The prognosis is abysmal once cervical cancer progresses to lymphatic metastasis. Anoikis, a specialized form of apoptosis induced by loss of cell adhesion to the extracellular matrix, plays a critical role. The prediction model based on anoikis-related genes (ARGs) expression and clinical data could greatly aid clinical decision-making. However, the relationship between ARGs and CESC remains unclear.

**Methods:**

ARGs curated from the GeneCards and Harmonizome portals were instrumental in delineating CESC subtypes and in developing a prognostic framework for patients afflicted with this condition. We further delved into the intricacies of the immune microenvironment and pathway enrichment across the identified subtypes. Finally, our efforts culminated in the creation of an innovative nomogram that integrates ARGs. The utility of this prognostic tool was underscored by Decision Curve Analysis (DCA), which illuminate its prospective benefits in guiding clinical interventions.

**Results:**

In our study, We discerned a set of 17 survival-pertinent, anoikis-related differentially expressed genes (DEGs) in CESC, from which nine were meticulously selected for the construction of prognostic models. The derived prognostic risk score was subsequently validated as an autonomous prognostic determinant. Through comprehensive functional analyses, we observed distinct immune profiles and drug response patterns among divergent prognostic stratifications. Further, we integrated the risk scores with the clinicopathological characteristics of CESC to develop a robust nomogram. DCA corroborated the utility of our model, demonstrating its potential to enhance patient outcomes through tailored clinical treatment strategies.

**Conclusion:**

The predictive signature, encompassing nine pivotal genes, alongside the meticulously constructed nomogram developed in this research, furnishes clinicians with a sophisticated tool for tailoring treatment strategies to individual patients diagnosed with CESC.

## 1 Introduction

Cervical carcinoma ranks as the fourth most common malignancy among women on a global scale (1). Data sourced from the Global Cancer Observatory, encompassing 185 countries, indicated that in 2018, there were approximately 570,000 active cases and 311,000 fatalities attributable to cervical cancer (2). Notably, breast and cervical carcinomas are among the trio of cancers witnessing a significant surge in incidence among women, particularly in developing nations. Furthermore, China and India collectively bear over a third of the worldwide cervical cancer burden (3). In developed countries, due to the popularization of Pap smear screening, the incidence of cervical cancer has been dramatically reduced (4).

Chronic infection with high-risk human papillomavirus (HPV) is recognized as a primary risk factor for cervical cancer development, with ≥90% of such malignancies being associated with high-risk HPV types (5, 6). Early-stage cervical cancer patients typically achieve reasonable local control yet are prone to distant recurrence, maintaining an overall survival rate of approximately 90%. Conversely, individuals with locally advanced cervical cancer face a higher risk of both distant and local failure, evidenced by a mortality rate of roughly 35% (7–9). Consequently, there is an imperative need for the identification of novel biomarkers that can accurately predict the prognosis of early-stage CESC, thereby facilitating timely and effective clinical interventions (10).

Anoikis refers to a distinct form of programmed cell death precipitated by cellular detachment from the extracellular matrix or neighboring cells (11). This process is pivotal in maintaining tissue homeostasis and preventing anomalous cell growth and attachment in inappropriate locations. Studies have found that most tumor cells have anti-apoptotic properties, whereby pro-apoptotic proteins are inhibited, the internal and external pathways of apoptosis are blocked, and cell survival factors are upregulated, which collectively facilitate the survival, invasion, and metastasis of tumor cells (12). However, research on the relationship between anoikis and distant metastasis in CESC is insufficient.

In this investigation, we explored the prognostic significance of ARGs in CESC and formulated an ARGs-based prognostic scoring model. Additionally, we executed a comprehensive examination of the variations within the tumor microenvironment (TME) and specifically scrutinized the immune-related aspects of the TME based on the risk score categorization.

## 2 Methods

### 2.1 Gene expression profile acquisition and patient data analysis

Gene expression profiles were meticulously selected based on predefined criteria from two distinct sources: 300 samples of CESC tissues were obtained from the Gene Expression Omnibus (GEO: GSE44001), and an additional 304 profiles, inclusive of three samples from normal adjacent tissues, were sourced from The Cancer Genome Atlas (TCGA-CESC). The inclusion criteria for these datasets were as follows: The cohort size needs to be large enough, including patients of different ages and stages, regardless of race, to be included in this study. All data underwent standardized normalization and batch effect removal processes to ensure consistency and reliability in subsequent analyses.

### 2.2 Anoikis-related gene identification

A comprehensive dataset of 514 ARGs was acquired from the GeneCards database (https://www.genecards.org/) and the Harmonizome portal, referenced in publications (13, 14). Following the initial data acquisition, a comprehensive analysis was performed on the TCGA-CESC cohort using the ‘limma’ package (Version: 3.58.1), a component of the Bioconductor software suite (Version: 3.18). This analytical process resulted in the identification of 170 differentially expressed genes (DEGs), achieved through a comparative evaluation of the expression profiles of the 514 ARGs between CESC tumor tissues and adjacent normal tissues.

### 2.3 Harmonized gene cluster analysis

To elucidate distinct patterns in anoikis regulation, we employed an advanced consensus clustering algorithm, utilizing the k-means method to categorize anoikis-related gene expression profiles. Subsequently, sophisticated techniques for dimensionality reduction were used, specifically t-distributed Stochastic Neighbor Embedding (t-SNE) and Uniform Manifold Approximation and Projection (UMAP). These methods facilitated the validation of the clustering’s robustness and reliability, utilizing the comprehensive capabilities of the R package ‘ggplot2’ for intricate data visualization and analysis.

### 2.4 Gene functional enrichment analysis

Gene Set Variation Analysis (GSVA) was chosen for its ability to provide a comprehensive assessment of gene set enrichment variations within CESC tissues, offering insights into the underlying molecular mechanisms. The gene set file ‘c2.cp.kegg.v7.4.symbols.gmt’ required for this analysis was obtained from the Molecular Signatures Database (MSigDB). The enrichment analysis was then meticulously executed using the ‘GSVA’ R package (version 1.50.0). Subsequently, the enrichment analysis was meticulously executed using the ‘GSVA’ R package (version 1.50.0), ensuring a rigorous and precise evaluation of the gene sets (15).

### 2.5 Elaboration and verification of anoikis-related prognostic signatures

The patient cohort was systematically partitioned into two distinct groups: a training set for the initial construction of the risk model and a validation set dedicated to its subsequent verification. Initial identification of genes associated with survival outcomes commenced with univariate Cox regression analysis. Subsequently, a refined analysis employing a minor absolute shrinkage and selection operator (LASSO) regression technique was performed, utilizing the ‘glmnet’ package (Version: 4.1–8) in R for implementation (16). Determination of the optimal penalty regularization parameter λ was achieved through an exhaustive 10-fold cross-validation methodology. In the ensuing stage, a sophisticated multivariate Cox regression approach was employed to delineate essential genes and ascertain their respective coefficients meticulously. A selection of nine pivotal anoikis-associated gene signatures was made to formulate the risk signatures, guided by the most favorable lambda values and their corresponding coefficients. The computation of each patient’s ARG signature risk score was $\text{RiskScore}=\sum_{1}^{9}Coefi*Expi$ .

In this model, ‘Coefi’ denotes the risk coefficient for each gene, while ‘Expi’ represents the gene’s expression level. To ascertain the predictive efficacy of this model, we utilized Kaplan-Meier (KM) survival analysis and time-dependent Receiver Operating Characteristic (ROC) curves. The dual approach facilitated a thorough evaluation of the model’s aptitude in forecasting patient outcomes. Then, a set of nine anoikis-related DEGs exhibiting a significant correlation with overall survival (OS) were discerned through meticulous multivariate Cox regression and LASSO analyses within both the GSE44001 and TCGA-CESC cohorts.

### 2.6 Analysis of risk score and immune cell correlation

In this research, the CIBERSORT algorithm and single-sample Gene Set Enrichment Analysis (ssGSEA) R scripts were employed for the quantitative assessment of the relative abundances of immune cell subsets infiltrating the tumor microenvironment (17). Specifically, CIBERSORT was utilized to ascertain the composition of various immune cell types within the groups with lower and higher prognostic risk. This method guaranteed that the aggregate score attributed to all inferred immune cell types in each specimen was normalized to one. Additionally, Spearman’s rank correlation methodology was utilized to elucidate the relationships between the computed risk scores and the degrees of immune cell infiltration.

### 2.7 Formulation and appraisal of predictive nomogram

An intricately crafted predictive nomogram integrating clinicopathological features with risk scores has been developed. A calibration plot is employed for internal validation purposes to enhance the model’s accuracy. Additionally, the nomogram’s prognostic capabilities were verified through the application of a time-cumulative index, providing a robust measure of its predictive performance. Furthermore, a decision curve analysis (DCA) was conducted meticulously to evaluate the net clinical benefits of the nomogram (18).

### 2.8 Tumor immune microenvironment single-cell analysis

The Tumor Immune Single-Cell Hub (TISCH; http://tisch.comp-genomics.org), a comprehensive online repository specializing in scRNA-sequencing data pertinent to the TME, was utilized as a pivotal resource in this study (19). Subsequently, Hallmark analysis was employed to assess apoptosis level changes within various cell clusters. This extensive database facilitated a systematic exploration of the heterogeneity within the TME across diverse datasets and cellular phenotypes.

### 2.9 Protein expression verification using human protein atlas

To meticulously corroborate the differential expression of the nine selected genes, including BCL2, BAX, IGF1, PLAU, EDA2R, ABL1, MIR200A, FASN, and NTRK3, in control versus cervical cancer tissues, immunohistochemical staining data from the Human Protein Atlas (HPA, http://www.proteinatlas.org) were systematically analyzed (20). This approach enabled a comprehensive visualization of the distinct expression patterns of these genes in tumorous tissue compared to normal tissue. The immunohistochemical analysis provided clear evidence of aberrant gene expression in the cancerous specimens, thereby substantiating the potential role of these genes in cervical carcinogenesis.

### 2.10 Cell culture

The Ect1/e6e7 and HeLa cell lines used in this study were purchased from the Shanghai Cell Bank (Shanghai, China). The cells were cultured in a complete medium (DMEM + 10% fetal bovine serum + 1% penicillin-streptomycin) and maintained in a 37°C incubator with 5% CO_2_ and saturated humidity. The medium was refreshed every 2–3 days. Cells were passaged when they reached over 90% confluence.

### 2.11 Reverse transcriptase-quantitative polymerase chain reaction

Cells were first rinsed with PBS after discarding the culture medium to prepare for total RNA extraction. RNA was isolated using the Trizol reagent (AG21102, Precision Biotechnology). The cDNA synthesis was performed with the Evo M-MLV RT Reverse Transcription Kit II (AG11711, Accurate Biotechnology). RT-qPCR was carried out using the SYBR Green Pro Taq HS premixed qPCR kit (AG11701, Accurate Biotechnology) on a LightCycler^®^ 96 system (Roche Ltd, Switzerland). Glyceraldehyde-3-phosphate dehydrogenase (GAPDH) served as the internal control. Gene expression fold changes were calculated using the 2^-ΔΔCT^ method. Primer sequences are listed in **Table 1**.

**Table 1:** Specific primers for reference and target genes.

Gene	Sense (5’-3’)	Anti-sense (5’-3’)
GAPDH	TGACCACAGTCCATGCCATCAC	CGCCTGCTTCACCACCTTCTT
ABL1	CGCTGAGTATCTGCTGAG	CACCGTTGAATGATGATGAA
BAX	CAGGATGCGTCCACCAAGA	CAGTTGAAGTTGCCGTCAGAA
BCL2	ACTTCGCCGAGATGTCCAG	TCCCAGCCTCCGTTATCCT
EDA2R	TCAATCGTGTTCAGAAGGTCAA	GCTCAACTGGAAGGCACATT
FASN	GGCATCCTGGCTGACGAAGACT	AGGTGCTGCTGAGGTTGGAGAG
IGF1	CCTCCTCGCATCTCTTCTACCT	GCAATACATCTCCAGCCTCCTT
NTRK3	TGCCTGTGTCCTGTTGGTGGTT	CGTGGTGATGCCGTGGTTGATG
PLAU	GGTCGCTCAAGGCTTAACTCCA	TCAGCAAGGCAATGTCGTTGTG

### 2.12 Validating the accuracy of prognostic indicators using the GEPIA data platform

Gene Expression Profiling Interactive Analysis (GEPIA) (http://gepia.cancer-pku.cn/) has RNA sequencing data of tumor tissues and normal tissues from TCGA and Genotype-Tissue Expression Project (GTEx), through which the platform not only allows access to gene expression in different tumors but also allows single-gene survival analysis (21). TCGA tumors vs TCGA normal + GTEx normal were compared using one-way ANOVA with a p-value cutoff of 0.05.

### 2.13 Comprehensive statistical methodology

Comprehensive statistical analyses were conducted utilizing R software (Version: 4.3.2). Specific statistical tests, including t-tests and ANOVA, were applied to meticulously analyze the data. The threshold for statistical significance was established at a P value of less than 0.05, and the false discovery rate (FDR) was rigorously controlled with a cut-off set at q<0.05. The experimental data are presented as mean ± standard deviation.

## 3 Results

### 3.1 Identification of prognosis-related ARGs in CESC

**Figure 1:**
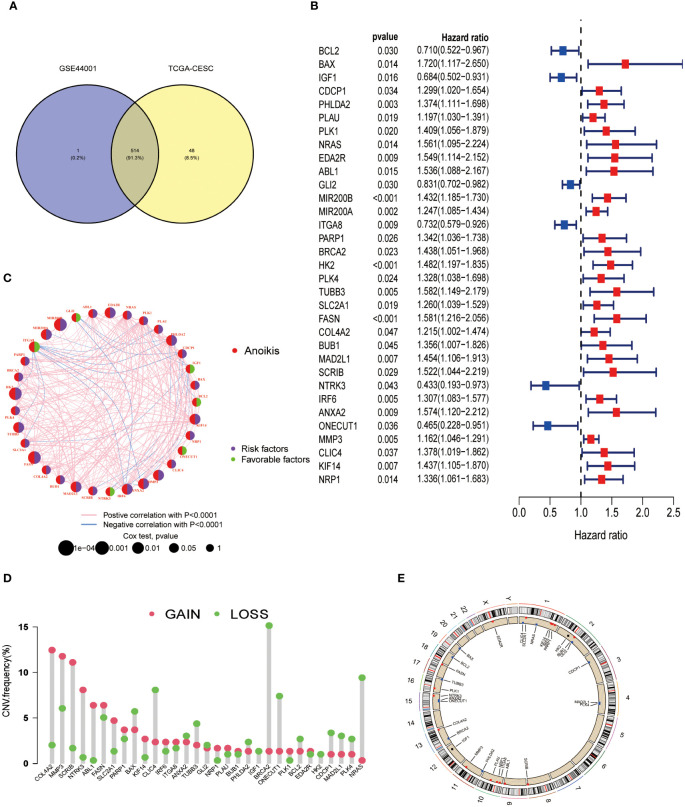
Differentially expressed ARGs in CESC and their correlation with prognosis were identified. **(A)** Identification of 514 genes associated with anoikis from study GSE44001 and the TCGA-CESC dataset. **(B)** A forest plot illustrating the most significant 33 ARGs (P < 0.05) identified through univariate Cox regression analysis. **(C)** A network diagram depicting the interrelationships among the top 33 ARGs. **(D)** Analysis of copy number variations (CNVs) in the 33 ARGs within the TCGA-CESC cohort. **(E)** Examination of chromosomal locations and alterations in the ARGs.

Initially, 563 ARGs were identified from the GeneCards and Harmonizome portals and narrowed down to 514 ARGs through Venn diagram analysis (**Figure 1A**). Comparison with normal adjacent tissues revealed 170 DEGs in CESC samples. Data from the TCGA-CESC cohort and GSE44001 were integrated, forming a consolidated ‘CESC-GSE44001’ cohort with 17,073 genes. Univariate Cox regression analysis identified 33 out of these 170 DEGs as significantly associated with patient survival (P < 0.05) (**Figure 1B**). Apart from BCL2, IGF1, GLI2, ITGA8, NTRK3, and ONECUT1, which indicated varied prognoses, the remaining 27 genes were predominantly correlated with poor prognosis. A network diagram revealed tight interconnections among these 33 genes (**Figure 1C**). Considering the frequent chromosomal aberrations in CESC, copy number variation data was extracted from the TCGA database (**Figure 1D**). Chromosomal changes in ARGs were thoroughly analyzed to locate each gene accurately. Chromosome one exhibited the highest occurrence of ARGs, including CLIC4, SLC2A1, KIF14, NRAS, IRF6, and PARP1. A significant genomic ‘GAIN’ was observed in COL4A2 on chromosome 13, coupled with a notable ‘LOSS’ of the BRCA2 gene on the same chromosome (**Figure 1E**).

### 3.2 Molecular subgroup distinctions in CESC via clustering of 33 ARGs

**Figure 2:**
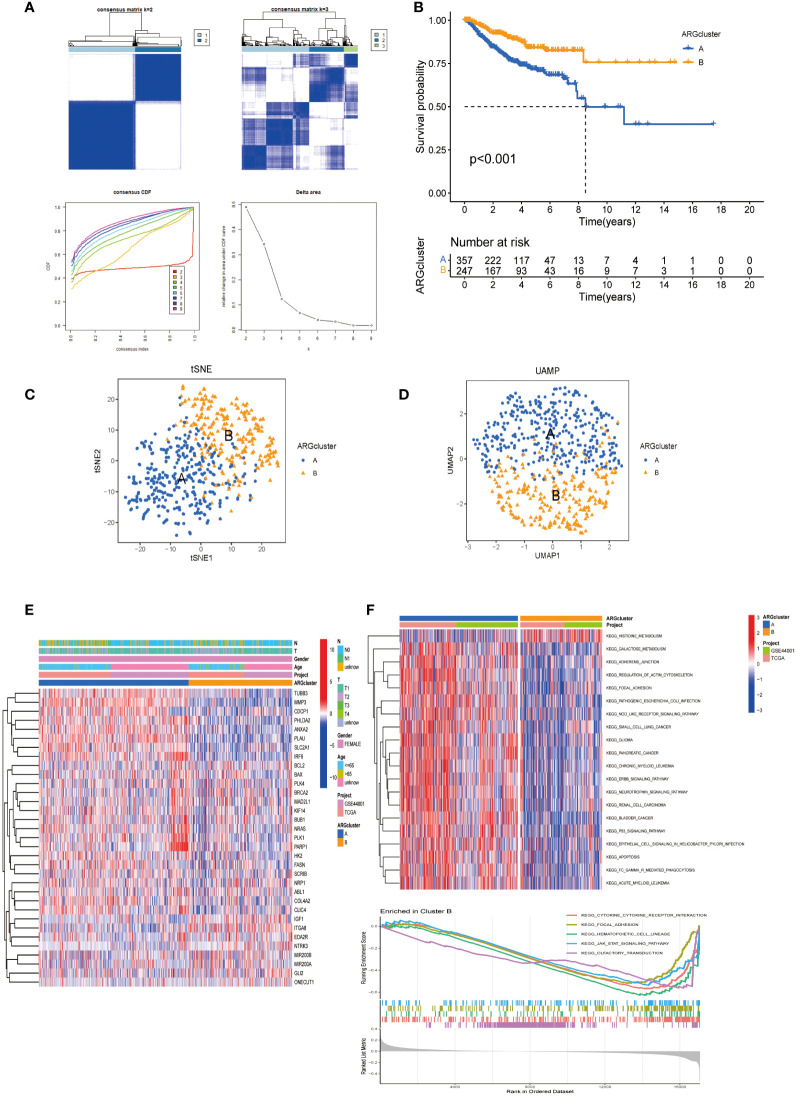
ARGs-based subgroup categorization in CESC. **(A)** A consensus matrix was established for k=2, utilizing consensus clustering. **(B)** The analysis of overall survival revealed significant differences between the two identified subgroups (P < 0.001). **(C, D)** Employing tSNE and UMAP techniques, the two subtypes were discerned based on ARG expression patterns. **(E)** A comprehensive heat map displayed the expression profiles of ARGs alongside the clinicopathological attributes of the two subtypes. **(F)** GSVA was conducted to pinpoint the distinct KEGG pathway enrichments contrasting between cluster B and cluster A.

To elucidate the functional implications of ARGs in CESC, we utilized the Consensus Cluster Plus R package (Version: 1.66.0) to perform consensus clustering of 33 prognosis-related DEGs (P <0.05). Optimal stratification into two distinct subtypes was achieved at k = 2 (**Figure 2A**). Subsequent overall survival (OS) analysis (P < 0.001) revealed a statistically notable prognostic disparity between these subtypes (**Figure 2B**). The validity of this bifurcation was corroborated through t-SNE and UMAP analyses, confirming the distinct segregation at k = 2 (**Figures 2C, D**). Heat maps delineating ARGs expression across these subtypes, alongside their clinicopathological attributes, indicated ITGA8 as a potential prognostic enhancer (**Figure 2E**). Furthermore, KEGG pathway enrichment analysis conducted for both subgroups identified significant enrichment of the histidine metabolism pathway in subgroup B, which exhibited a favorable prognosis (**Figure 2F**). Additionally, Gene Set Enrichment Analysis (GSEA) underscored reduced cytokine-cytokine receptor interactions, enrichment of focal adhesion, and JAK-STAT signaling pathways in subgroup B, pivotal pathways implicated in tumor cell migration and colonization of new anchorage sites (**Figure 2F**).

### 3.3 Differential gene expression and immune cell infiltration patterns in subgroups

**Figure 3:**
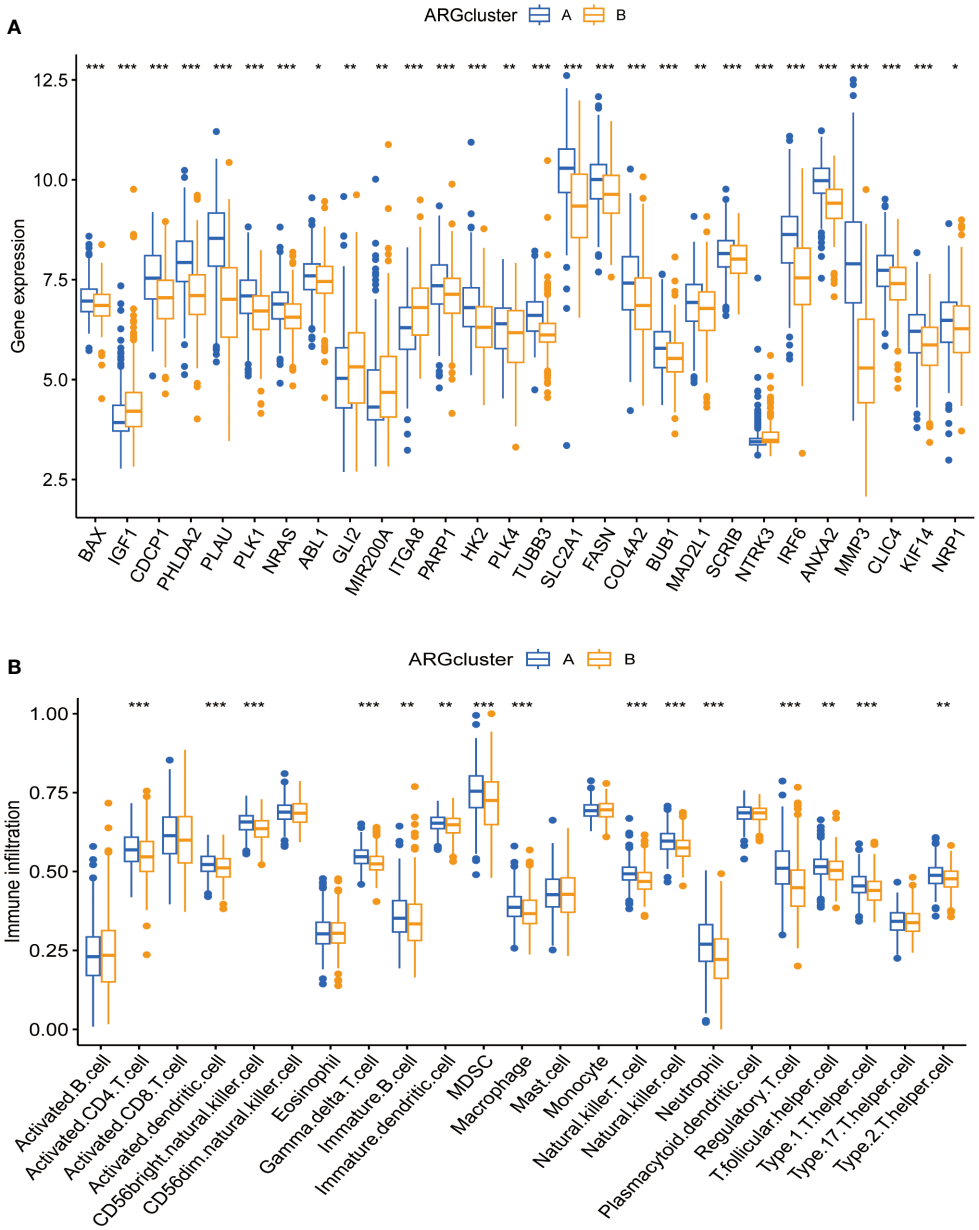
Expression patterns of ARGs and immune infiltration in distinct subtype clusters. **(A)** Analysis of ARGs expression across the two different subtype clusters. **(B)** Examination of immune infiltration characteristics within these two subtype clusters. Significance: *P < 0.05, **P < 0.01, ***P < 0.001.

Box plots show the expression patterns of ARGs in the two subgroups (**Figure 3A**). IGF1, GLI2, MIR200A, ITGA8, and NTRK3 levels were lower in subgroup A than in subgroup B. Conversely, the other genes were highly expressed in subgroup A. Given their correlation with overall survival, these DEGs may encode pivotal molecules that significantly influence the prognosis of patients with CESC. Furthermore, they present as potential targets for developing targeted therapeutic strategies. There were also significant differences in the extent of immune cell infiltration, and the proportions of activated CD4^+^ T cells, dendritic cells, and other immune cells in subgroup A were significantly higher than those in subgroup B (**Figure 3B**).

### 3.4 Construction and validation of prognostic signals related to anoikis

**Figure 4:**
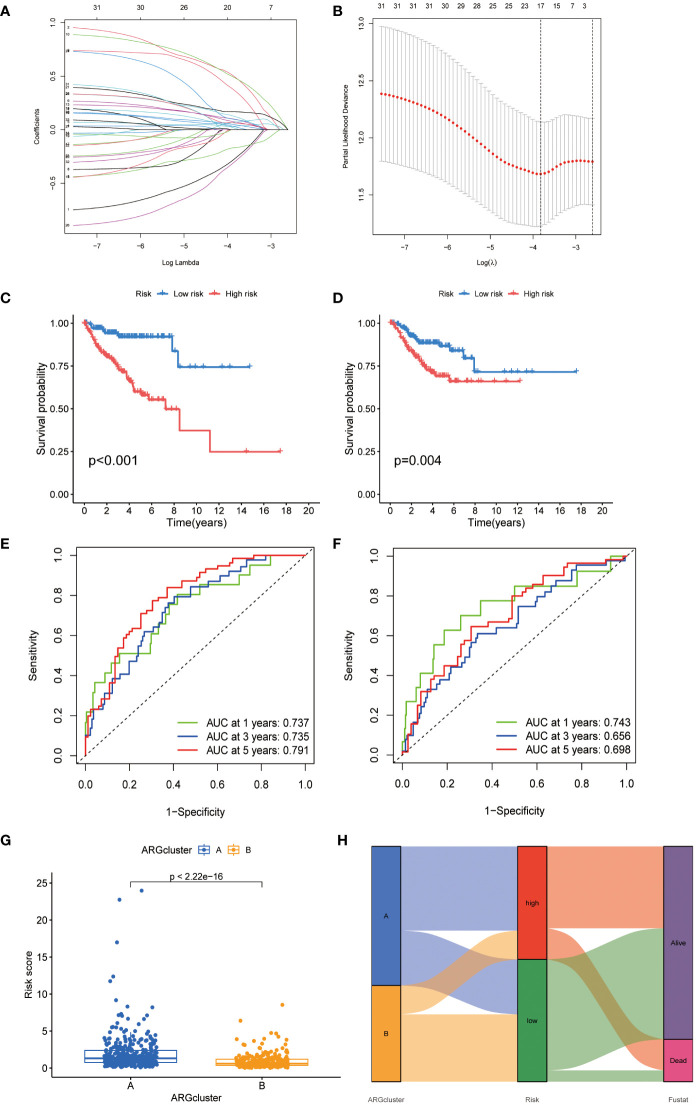
Development of an ARGs-based prognostic signature. **(A)** Identification of prognostic ARGs using LASSO regression with 10-fold cross-validation. **(B)** Visualization of coefficients for seventeen key prognostic ARGs. **(C, D)** Kaplan-Meier curves showing survival variations in different risk subtypes. **(E, F)** Time-dependent ROC analysis for OS at 1, 3, and 5 years. **(G)** Risk score evaluation in two established clusters. **(H)** Alluvial diagram depicting transitions between subtypes and survival status.

In our investigation into the clinical relevance of ARGs, 33 ARGs (p < 0.05) were subjected to Lasso-penalized Cox regression analysis (**Figures 4A, B**). The optimal lambda (λ) value for Lasso regression, corresponding to the minimum cross-validation error, was determined to be 17. To refine our predictive model, multivariate Cox regression was employed, leading to the selection of 9 pivotal ARGs from the initial 17. These were instrumental in constructing the prognostic model. The resultant risk score, based on this nine-ARG signature, was designated as the “ARG score” with corresponding correlation coefficients detailed in **Supplementary Table S1**. The Prognostic Index (PI) was calculated as follows: PI = (0.929 × BAX expression) + (0.177 × PLAU expression) + (0.729 × EDA2R expression) + (0.782 × ABL1 expression) + (0.197 × MIR200A expression) + (0.317 × FASN expression) − (0.616 × BCL2 expression) − (0.362 × IGF1 expression) − (0.978 × NTRK3 expression).

Kaplan-Meier analysis indicated a poorer prognosis for patients in the high-risk group, a trend corroborated in the TCGA-CESC validation cohort (**Figures 4C, D**). Time-dependent ROC curves for the model, assessing 1-, 3-, and 5-year overall survival, demonstrated its robust predictive accuracy (**Figures 4E, F**). A significant disparity in risk scores was observed between the previously identified subtypes (**Figures 4G, H**). An alluvial diagram was constructed to illustrate transitions among ARG clusters, changes in ARG scores, and survival status.

### 3.5 Gene set enrichment analysis and immune activity with the different risk score

**Figure 5:**
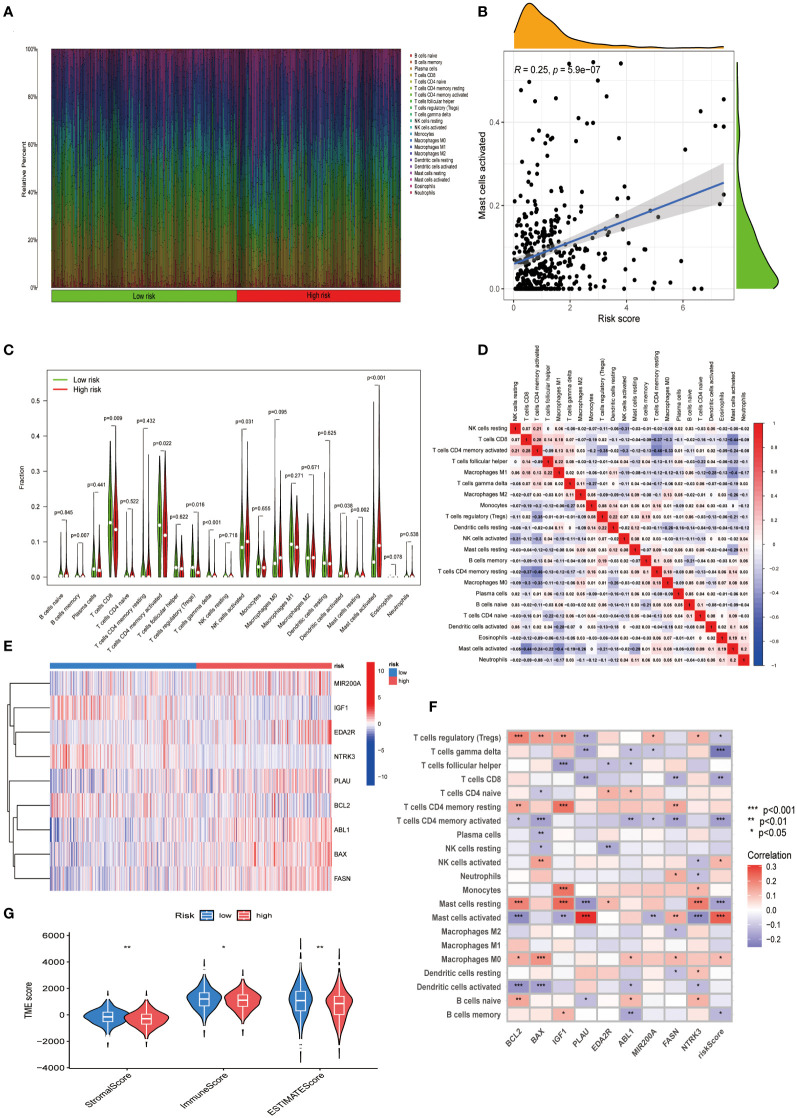
Exploring the immune landscape in CESC’s TME across varying risk scores. **(A)** Analysis of the composition of immune cell infiltration at different risk levels. **(B)** Investigating the relationship between risk scores and the abundance of activated Mast cells in CESC. **(C)** Comparison of immune cell profiles in high-risk and low-risk groups. **(D)** Examination of interrelations among different immune cells. **(E)** A heatmap presenting the expression patterns of nine central ARGs. **(F)** Study of the associations between immune cells and these nine pivotal ARGs. **(G)** Comparative analysis of estimate scores based on expression profiles in high-risk versus low-risk groups. Significance: *P < 0.05, **P < 0.01, ***P < 0.001.

We investigated the differences in the TME of CESC patients classified into high- and low-risk categories. We conducted a quantitative analysis of immune cell proportions using CIBERSORT R Scripts, ordering CESC samples from lowest to highest risk scores to reflect the variability in immune cell types (**Figure 5A**). Notably, a rising trend in activated mast cells correlated with increasing risk scores (R = 0.25) (**Figure 5B**). Additionally, eight other immune cell types showed correlations with risk scores (**Supplementary Figure S1**). In high-risk patients, activated mast cells constituted a more significant portion of the TME (**Figure 5C**), suggesting their influence on adverse prognoses. This association of various immune cells with CESC risk categories enhances our understanding of TME composition in these tumors (**Figure 5D**). The nine-gene ARG score model demonstrated distinct expression patterns between risk groups, corresponding to variations in immune cell infiltration (**Figures 5E, F**). Differential stromal and immune scores, derived from expression profile evaluations, were observed between the high- and low-risk groups (**Figure 5G**). Moreover, using the ‘oncoPredict’ R package (Version: 0.2), we identified differential drug susceptibility profiles across these groups, highlighting potential therapeutic avenues (**Supplementary Table S2**; **Supplementary Figures S2, S3**).

### 3.6 Establishment of a prognostic nomogram with high efficacy

**Figure 6:**
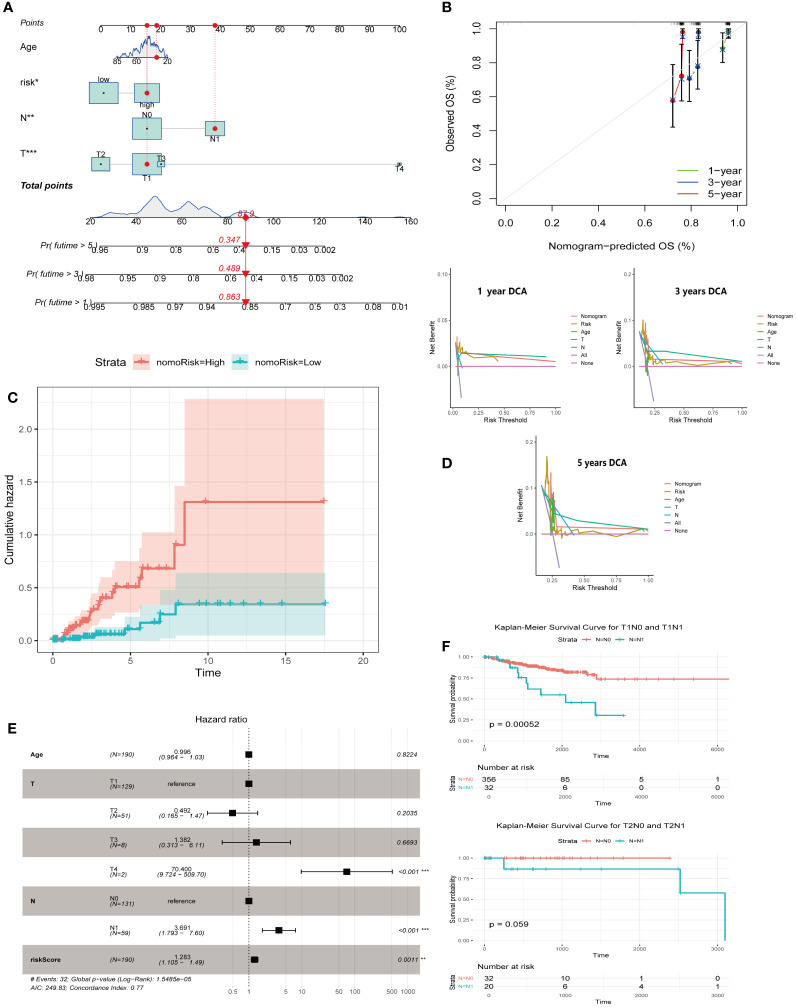
Creation of a nomogram for predicting outcomes in CESC. **(A)** Construction of a nomogram integrating ARG-score with various clinicopathological parameters. **(B)** A calibration curve is employed to verify the accuracy of the nomogram. **(C)** A cumulative hazard curve depicting survival probabilities over time for patients. **(D)** DCA is used to evaluate the nomogram’s effectiveness at 1, 3, and 5 years for OS in CESC. **(E)** A forest plot summarizing the outcomes of multivariable Cox regression analyses, including clinical features and risk scores in CESC. **(F)** Survival analyses of T1N0 vs. T1N1, T2N0 vs T2N1. Significance: *P < 0.05, **P < 0.01, ***P < 0.001.

We integrated the ARG score with the clinical and pathological characteristics of CESC patients to develop a comprehensive nomogram (**Figure 6A**). The accuracy and prognostic efficacy of this nomogram were confirmed by the calibration plot (**Figure 6B**). The cumulative risk curve (**Figure 6C**) showed a progressive increase in overall survival risk for CESC patients with higher nomogram scores, underscoring its prognostic value. Decision Curve Analysis demonstrated the ability of the nomogram to predict short- and long-term survival outcomes in CESC patients (**Figure 6D**). The forest plot identified the T-stage, N-stage, and risk score as crucial risk factors within the nomogram (**Figure 6E**). Survival analyses comparing T1N0 vs. T1N1 and T2N0 vs. T2N1 indicated significantly lower survival rates for patients with N1 during T1 compared to N0 (P<0.001) (**Figure 6F**). These findings collectively validate our ARG-based nomogram as a robust tool for clinical prognosis in CESC patients, significantly contributing to personalized patient care strategies.

### 3.7 BAX and PLAU are closely associated with lower apoptosis levels in tumor cells

**Figure 7:**
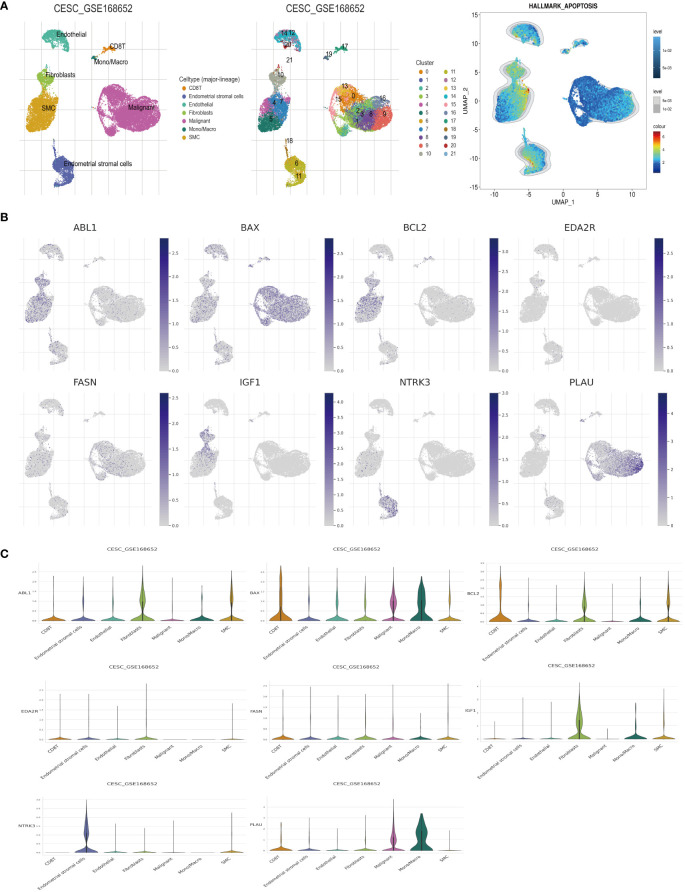
Analysis of ARGs in CESC’s TME cells using scRNA-seq database. **(A)** Cell type identification, quantification, and Hallmark analysis in dataset GSE168652. **(B, C)** Analysis of the percentages and expression levels of crucial ARGs, including ABL1, BAX, BCL2, EDA2R, FASN, IGF1, NTRK3, and PLAU.

Mapping the expression of anoikis-associated genes to different cell types in the tumor microenvironment elucidated the interactions between these genes in tumor, stromal, and immune cells, crucial for understanding anoikis resistance and cancer progression. We analyzed the expression profiles of eight ARGs in the TME using the CESC_GSE168652 single-cell dataset from TISCH. This dataset identified 21 cell clusters across seven distinct cell types (**Figure 7A**). ABL1, BCL2, and IGF1 exhibited high expression in fibroblasts and smooth muscle cells (SMCs). BAX and PLAU were highly expressed in malignant cells, while FASN showed broad expression across multiple cell types. EDA2R had low expression in various cell types, whereas NTRK3 was highly expressed in endometrial stromal cells (**Figures 7B, C**). Hallmark analysis revealed lower apoptosis levels in tumor cells, marked by BAX and PLAU, indicating their unique roles (**Figure 7A**). Since MIR200A was not expressed in the cell clusters, it was excluded from the analysis.

### 3.8 Verification results of ARGs align with expression trends in the risk prediction model

**Figure 8:**
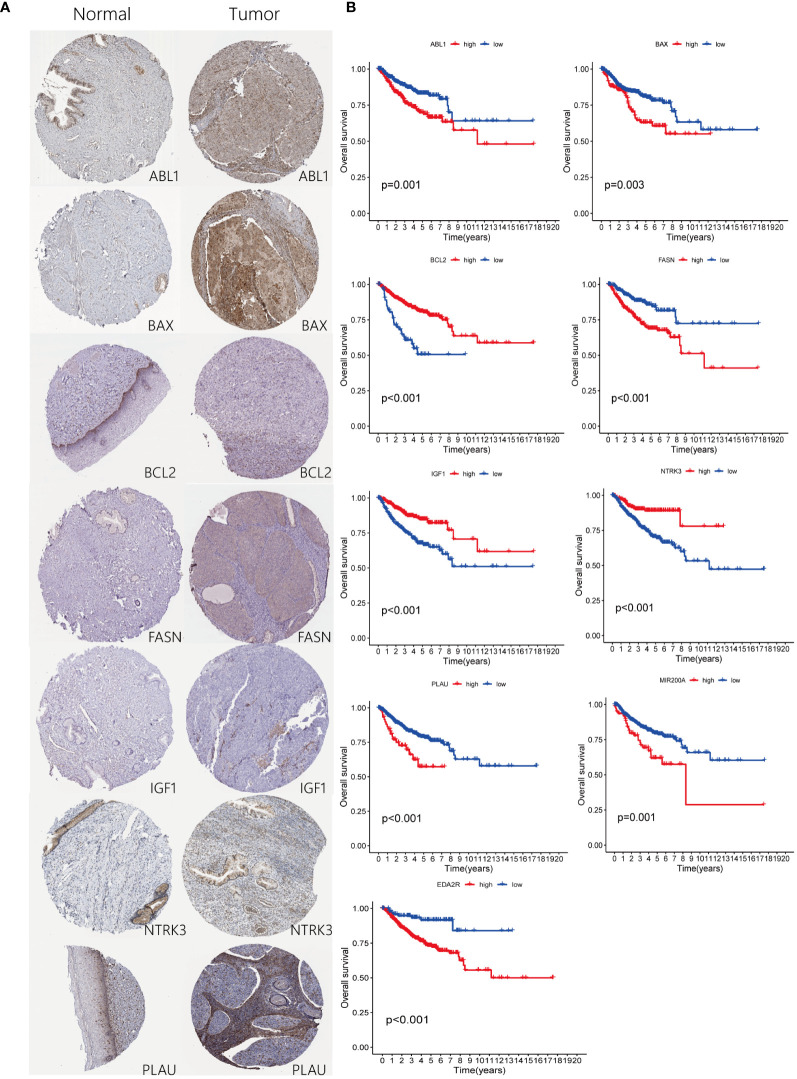
Expression of ARGs in risk-stratified groups and their prognostic significance. **(A)** Comparison of ARGs expression through immunohistochemical staining in both control and pathological tissues. **(B)** Prognostic implications of ARGs for patients categorized into high and low-risk groups.

**Figure 9:**
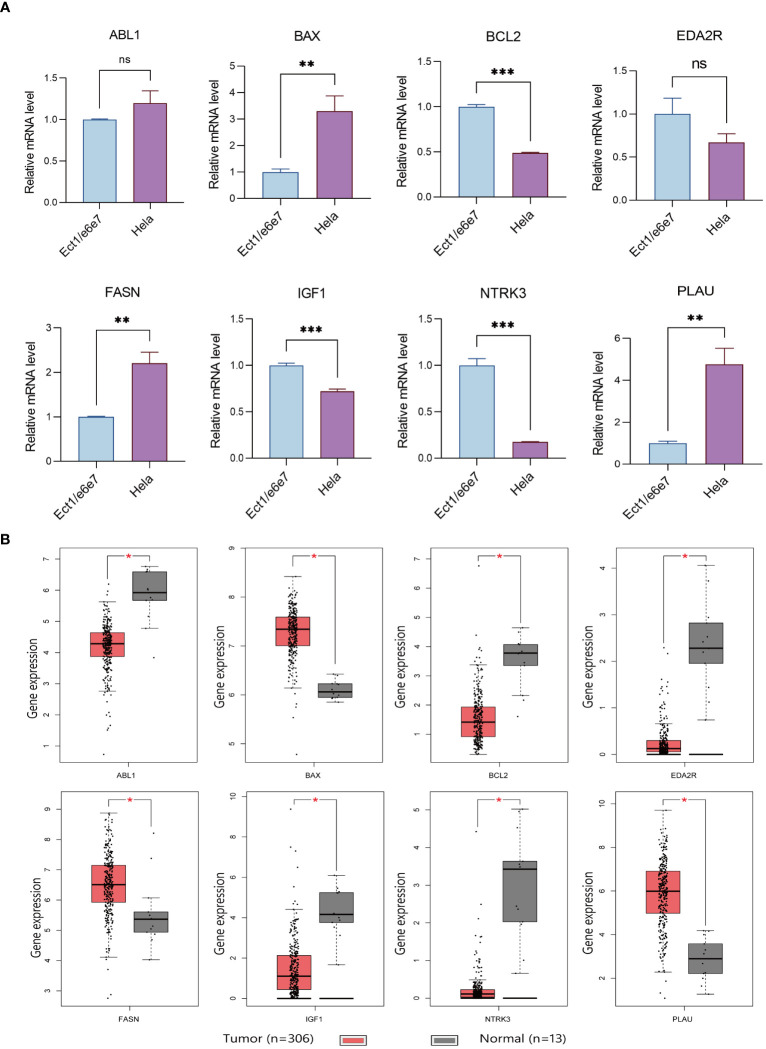
Verification results of ARGs. **(A)** Comparison of ARGs mRNA expression in normal and tumor cell lines using RT-qPCR (with GAPDH as the internal control). * p<0.05, **p<0.01, ***p<0.001. Data are presented as mean ± SD. **(B)** Prognostic indicators expressed in TCGA tumors vs TCGA normal + GTEx normal. The “ns” means “no significance”.

Immunohistochemical analysis utilizing the HPA database revealed a notable disparity in gene expression within cervical cancer tissues. Specifically, the genes ABL1, BAX, FASN, and PLAU exhibited abnormally high expression levels in cervical cancer specimens, in stark contrast to BCL2, IGF1, and NTRK3, which were markedly under-expressed (**Figure 8A**). This differential expression pattern was further corroborated by survival analysis. The survival curves, based on the expression of each gene, provided additional evidence supporting the observed expression trends in relation to patient survival outcomes (**Figure 8B**). Further *in vitro* experiments indicated that, compared to Ect1/e6e7 cells, Hela cells exhibited upregulated mRNA levels of BAX, FASN, and PLAU, while BCL2, IGF1, and NTRK3 mRNA levels were downregulated. ABL1 and EDA2R did not show significant differences (**Figure 9A**). To validate these findings further, we analyzed GEPIA data, which showed similar results. Compared with normal tissue, BAX, FASN, and PLAU were significantly upregulated in CESC, but ABL1 and EDA2R did not achieve the expected results (**Figure 9B**). Meanwhile, BCL2, IGF1, and NTRK3 expression were significantly reduced in the tumors.

## 4 Discussion

In this study, we identified 33 differentially expressed ARGs associated with the prognosis of CESC and explored their clinical significance via consensus clustering. Analyzing these genes, we investigated the dynamic tumor microenvironment of CESC, particularly the immune milieu. Using Lasso-penalized Cox regression, we developed a prognostic model based on nine ARGs (BCL2, BAX, IGF1, PLAU, EDA2R, ABL1, FASN, NTRK3, and MIR200A), revealing significant clinical potential. This model demonstrated the association of these genes with the tumor immune microenvironment, notably T-cells and mast cells, and validated expression differences both *in vivo* and *in vitro*. Therefore, this model provides valuable insights for early screening, prognostic prediction, and clinical treatment of CESC.

Identifying specific biomarkers is crucial for early detection, prognosis, and personalized treatment (22). Understanding mechanisms like anti-apoptosis, cell invasion, and immune evasion is vital for developing targeted therapies. Although models based on ferroptosis, pyroptosis, and N6-methyladenosine have been proposed, their predictive efficacy is insufficient, necessitating new models (23–25). Anoikis serves as a natural barrier against metastasis by preventing the colonization of tumor cells at non-native sites. However, malignant tumor cells, mainly those capable of distant metastasis, often develop resistance to anoikis. They employ various strategies to circumvent this process, including the autocrine release of growth factors like IL-6, paracrine signaling with molecules such as VEGFa/VEGFR2, activation of pro-survival pathways like ERK and PI3K, and alterations in integrin expression patterns (26–28).

These adaptive mechanisms enable tumor cells to survive and thrive in new microenvironments, facilitating metastasis (29). Anoikis-based predictive models have broad applications. For instance, Tianlei X et al. (30) developed the four-gene feature “Ascore,” which demonstrated superior predictive capability for bladder cancer immunotherapy response, surpassing PD-L1. Additionally, Junyi L et al. (31) found that ARGs are closely associated with the drug resistance of clear cell renal cell carcinoma. The aberrant expression of ARGs is notably linked to the distant metastasis of cervical cancer, underscoring the need for more in-depth research in this area (32). In this study, ARGs-related nomograms demonstrated excellent prognostic predictive capability and were closely associated with the biological mechanisms of CESC development, consistent with previous research findings.

In the present study, we identified nine ARGs, including BCL2, BAX, IGF1, PLAU, EDA2R, ABL1, FASN, NTRK3, and MIR200A, which are closely associated with tumor development. The BCL2 protein family predominantly orchestrates the intrinsic apoptotic cascade, and perturbations within the TP53 pathway are frequently implicated in the oncogenesis of various tumors. Our findings substantiate this, revealing a conspicuous reduction in BCL2 expression within the high-risk cohort (33). Insulin-like growth factor-1 (IGF-1), essential for cellular proliferation, when inhibited, leads to significant shifts, including increased cellular accumulation at the G2M/S phase, augmented apoptosis, and reduced invasive capabilities of tumor cells (34). EDA2R mediates the activation of NF-κB and JNK pathways and is closely associated with cancer cachexia (35). Additionally, the ABL1 gene regulates cytoskeletal dynamics and is linked to tumor drug resistance and cell migration (36, 37). FASN encodes a fatty acid synthase that catalyzes the conversion of acetyl coenzyme A and malonyl coenzyme A to palmitate (38). Overexpression and hyperactivity of FASN are typically associated with malignant cells. Mutations in the NTRK gene, which encodes a member of the neurotrophic tyrosine receptor kinase (NTRK) family, promote myeloid medulloblastoma and secretory breast cancer (39). Although machine learning proved that these genes are closely related to CESC, further analytical biology experiments are needed to confirm their correlation with CESC.

In the present study, single-cell sequencing analysis revealed that BAX and PLAU are closely associated with lower apoptosis levels within CESC. BAX, a member of the pro-apoptotic protein family, is a crucial effector of mitochondrial apoptosis induced by most BH3 mimetics and chemotherapeutic agents (40). Recent studies have shown that co-targeting BAX and BCL-XL proteins can overcome cancer resistance to apoptosis (41). The present study indicated that high BAX expression is associated with poor prognosis, suggesting that using BAX activators might yield better clinical outcomes. Additionally, concurrent research has highlighted the pronounced upregulation of PLAU in cervical carcinoma cells. PLAU is known to be closely related to tumor diagnosis, therapeutic targeting, and patient prognosis (42). A recent study demonstrated that overexpressed PLAU in tumor tissues synergizes with FOXM1 to promote gastric cancer progression (43). Rigorous *in vitro* analyses have shown that targeted attenuation of PLAU expression significantly reduces the migratory and invasive capabilities of HeLa and HT3 cell lines. Furthermore, the core promoter of PLAU was delineated to reveal transcriptional regulation by YinYang 1 (YY1), a crucial modulator of PLAU mRNA expression (44). This study emphasizes the promising clinical applications of targeting BAX and PLAU in tumor cells. However, considering that the mechanisms of anoikis resistance primarily occur in distant metastatic tumors, our subsequent research will focus on single-cell sequencing results from both primary and distant metastatic tumors, which may provide more valuable insights.

Various methodologies for sample classification based on predefined gene expression profiles have been documented in the literature, employing diverse analytical techniques (45–47). In our investigation, we developed a nomogram predicated upon a selected array of ARGs, facilitating the stratification of patients into distinct prognostic categories. We observed marked variances in the expression of these ARGs among the identified subgroups, correlating significantly with patient prognoses. It underscores the efficacy of our nine-gene nomogram in prognostication, thereby aiding clinicians in devising tailored treatment strategies. Moreover, the DCA indicated that the nomogram, based on these nine genes, potentially offers clinical benefits to patients with CESC at one-, three-, and five-years post-diagnosis. Future research will aim to apply this model in clinical practice.

The study extended the examination to the TME, significantly influencing tumor metastasis and the efficacy of targeted therapies, building on the previously discussed nomogram-based classification (48). Our analysis included the infiltration levels of 21 immune cell types across different patient subtypes. High-risk subgroups with lower survival rates showed a significant increase in activated mast cell infiltration. Mast cells promote angiogenesis and neovascularization by releasing pro-angiogenic factors, including VEGF, FGF-2, PDGF, and IL-6, and non-classical factors like trypsin-like and chymotrypsin (49). This underscores their critical role in CESC progression. Among the nine identified risk genes, PLAU had the strongest correlation with activated mast cell levels, highlighting the PLAU/activated mast cell axis for further exploration. Additionally, severe dysregulation of the T-cell population, including decreased CD8^+^ T cells, was identified. Recent studies have shown that many patients do not benefit from immune checkpoint blockade therapy due to low CD8^+^ T cell infiltration in the TME (50). Furthermore, the specific biological mechanisms of ARGs and the TME, as well as whether targeted therapies can reduce tumor development, resistance, and distant metastasis, require more extensive *in vivo* studies, such as xenografts or allografts.

In conclusion, our study has successfully established a nine-gene model that exhibits remarkable accuracy in prognosticating outcomes for patients with CESC. The nomogram, derived from this model, serves as a valuable tool in clinical practice, enabling physicians to tailor personalized treatment strategies for CESC patients. However, it is crucial to delve deeper into the molecular mechanisms that underpin these gene signatures, particularly at the single-cell level, to gain a more comprehensive understanding of their role in tumor progression and patient prognosis. Furthermore, the expansion of our research to include larger patient cohorts and the conduction of prospective randomized clinical trials are imperative for validating and refining our model. Such endeavors will undoubtedly contribute significantly to the field of precision medicine, potentially leading to more effective and individualized therapeutic approaches for CESC patients.

## Data availability statement

The original contributions presented in the study are included in the article/**Supplementary Material**. Further inquiries can be directed to the corresponding author.

## Author contributions

M-W: Data curation, Formal analysis, Methodology, Visualization, Writing – original draft, Writing – review & editing. Q-Y: Investigation, Project administration, Validation, Writing – original draft, Writing – review & editing. RD: Methodology, Writing – original draft. Y-X: Validation, Writing – original draft. JW: Writing – review & editing. Y-P: Writing – review & editing. BP: Writing – review & editing. G-X: Writing – review & editing. ZL: Conceptualization, Funding acquisition, Resources, Supervision, Writing – review & editing.
