# Supplementary figures and images for: Elucidating prognosis in cervical squamous cell carcinoma and endocervical adenocarcinoma: a novel anoikis-related gene signature model
Source: Front Oncol. 2024 Jun 26;14:1352638. doi: 10.3389/fonc.2024.1352638 (PMC11234598; doi:10.3389/fonc.2024.1352638)

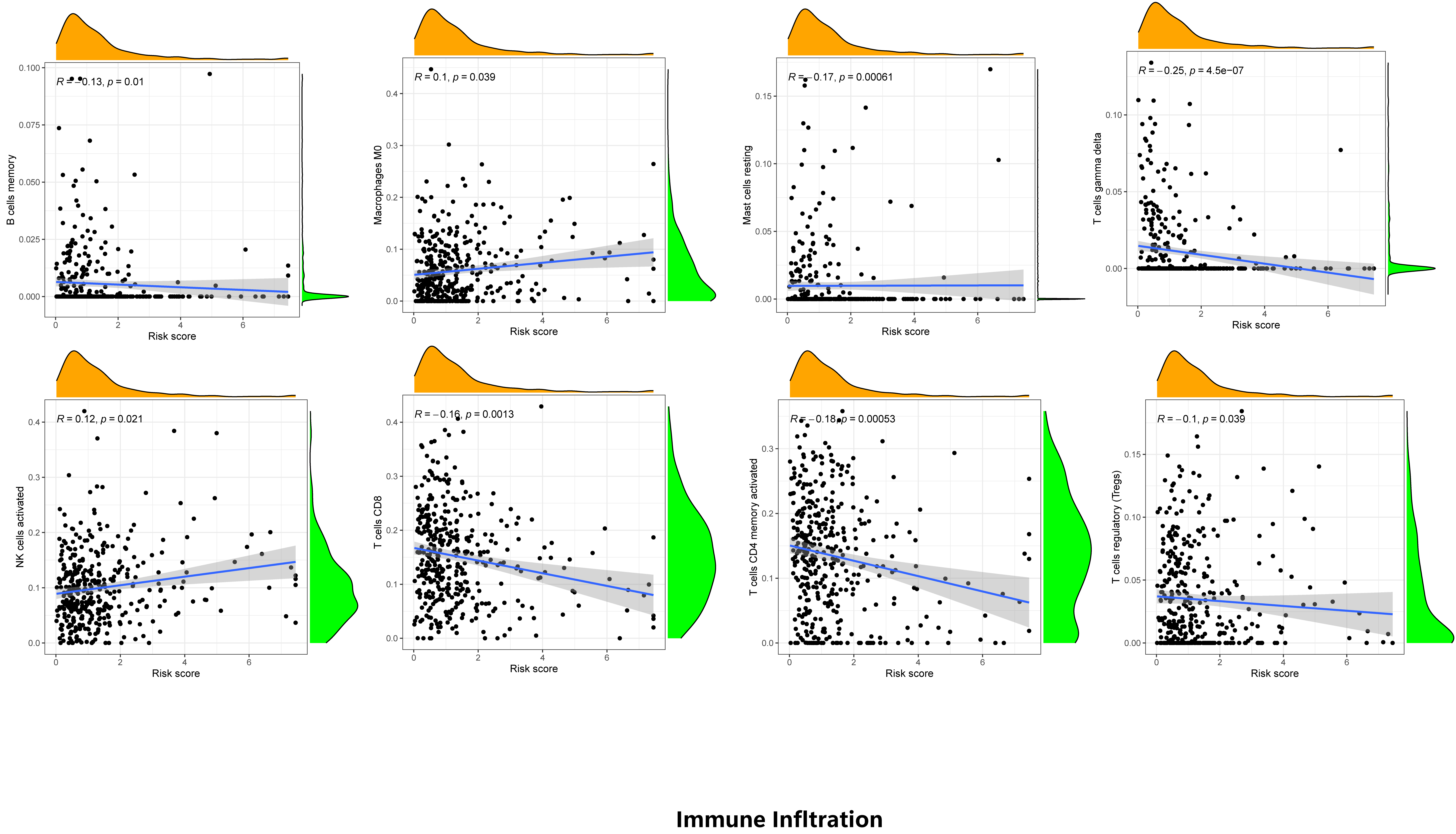

Supplement: Supplementary file 1 [file Image_1.tif]

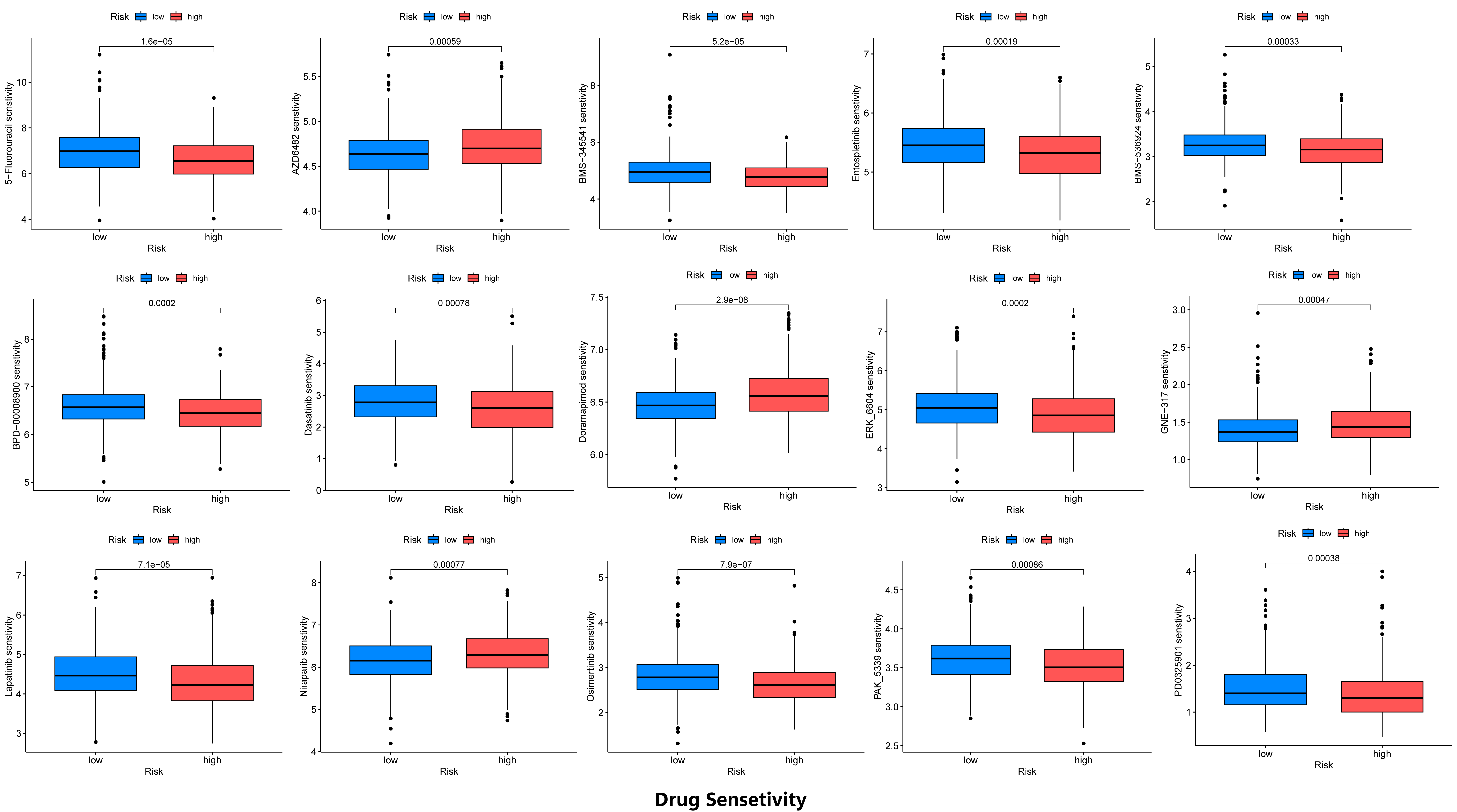

Supplement: Supplementary file 2 [file Image_2.tif]

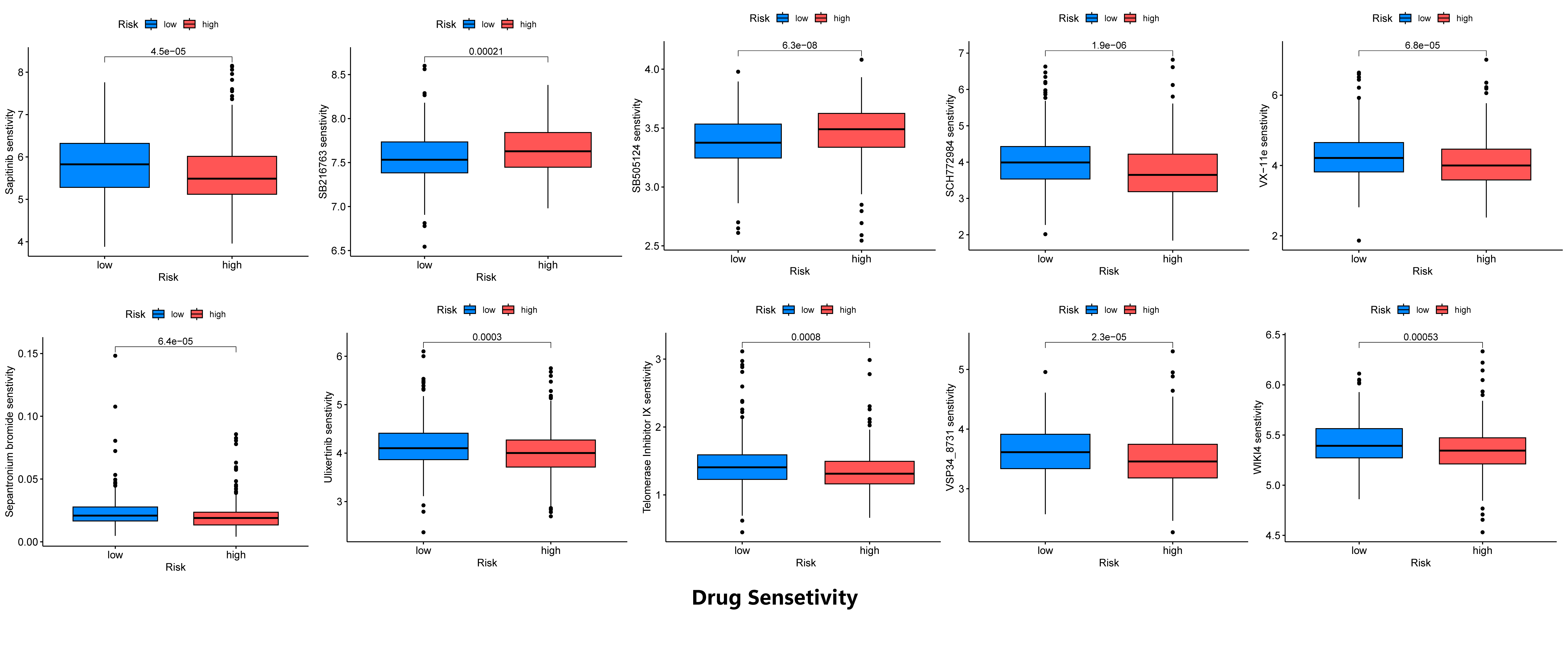

Supplement: Supplementary file 3 [file Image_3.tif]
